# Integration of probabilistic regulatory networks into constraint-based models of metabolism with applications to Alzheimer’s disease

**DOI:** 10.1186/s12859-019-2872-8

**Published:** 2019-07-10

**Authors:** Han Yu, Rachael Hageman Blair

**Affiliations:** State University of New York at Buffalo, 3435 Main Street, Buffalo, 14214 US

**Keywords:** Constraint-based model, Gene network, Bayesian network, Model integration, Probabilistic reasoning, Belief propagation, Metabolism

## Abstract

**Background:**

Mathematical models of biological networks can provide important predictions and insights into complex disease. Constraint-based models of cellular metabolism and probabilistic models of gene regulatory networks are two distinct areas that have progressed rapidly in parallel over the past decade. In principle, gene regulatory networks and metabolic networks underly the same complex phenotypes and diseases. However, systematic integration of these two model systems remains a fundamental challenge.

**Results:**

In this work, we address this challenge by fusing probabilistic models of gene regulatory networks into constraint-based models of metabolism. The novel approach utilizes probabilistic reasoning in BN models of regulatory networks serves as the “glue” that enables a natural interface between the two systems. Probabilistic reasoning is used to predict and quantify system-wide effects of perturbation to the regulatory network in the form of constraints for flux variability analysis. In this setting, both regulatory and metabolic networks inherently account for uncertainty. Applications leverage constraint-based metabolic models of brain metabolism and gene regulatory networks parameterized by gene expression data from the hippocampus to investigate the role of the HIF-1 pathway in Alzheimer’s disease. Integrated models support HIF-1A as effective target to reduce the effects of hypoxia in Alzheimer’s disease. However, HIF-1A activation is far less effective in shifting metabolism when compared to brain metabolism in healthy controls.

**Conclusions:**

The direct integration of probabilistic regulatory networks into constraint-based models of metabolism provides novel insights into how perturbations in the regulatory network may influence metabolic states. Predictive modeling of enzymatic activity can be facilitated using probabilistic reasoning, thereby extending the predictive capacity of the network. This framework for model integration is generalizable to other systems.

**Electronic supplementary material:**

The online version of this article (10.1186/s12859-019-2872-8) contains supplementary material, which is available to authorized users.

## Background

Advances in high-throughput technologies have made large-scale measurements of molecular traits possible. Mathematical and probabilistic models of networks have become instrumental in elucidating complex relationships among molecular traits from high-throughput data, e.g., [1–4]. However, networks often target specific domains of biological variables such as protein-protein interaction networks, metabolic networks and genetic networks. Data integration remains a major challenge for systems biology, especially at the network level, thereby limiting our ability to take full advantage of the wealth of post-genomics data.

This work describes a novel approach to network integration that aims to understand how gene regulatory networks influence metabolism. Our approach interfaces two network-based approaches that have evolved largely in parallel: (1) constraint-based models of cellular metabolism [5] and (2) Probabilistic Graphical Models (PGMs) of a gene regulatory networks [6]. These approaches have unique and complementary characterizations and predictive capabilities. Metabolic models do not reflect the individual variation in the fluxes that result from allelic variation of enzymes, or from regulation at the transcriptional level. On the other hand, methods for fitting PGMs often ignore all prior information about the biological pathway [7, 8]. Bridging these modeling strategies is a novel pursuit that may lead to more accurate physiological representations of cellular metabolism that account for genetic variability and differential regulation of the biochemical reactions. To authors knowledge, the integration of these two modeling paradigms has not been examined yet. Computational models of this nature are of fundamental importance for the prevention and treatment of disease.

Gene regulatory networks play an important role in fundamental processes such as cell cycle, differentiation and signal transduction and metabolism [8]. Understanding of the networks and the impact of their dysregulation can provide insights into processes and mechanisms underlying disease. In many cases, the structure of gene networks is not well understood, and a broad range of methods have been proposed to infer (aka reverse engineer) network structures from data (e.g., genomic, gene-expression and clinical phenotypes) [7–13]. Graphical models can be directed or undirected [11, 14, 15], indicating causality or association, respectively [16]. For example, directed networks have been used for time-series omics data, and also in genotype-phenotype network modeling [10, 17–23]. The appropriateness of a directed graphical model for causal interpretations depends on the data and experiment at hand [24]. Recently, Moharil et al. [25] described an approach to propagating information through a directed gene network as a way to predict the system-wide response of the network to genetic perturbations. The approach utilized belief prorogation in Bayesian Networks (BNs), and to our knowledge, is the first to shift focus from network structural inference, to the problem of post-hoc network analysis and in silico prediction. In this work, we leverage the belief propagation in BNs to provide an interface between genetic and metabolic networks.

Constraint-based modeling has been widely used in systems biology as a computational tool to provide insights into cellular metabolism [26, 27]. The underlying metabolic models describe a complex network of biochemical reactions governed by stoichiometry, laws of mass balance, environmental and regulatory constraints and do not rely on the specification of kinetic parameters [28, 29]. Several metabolic reconstructions have been published [27, 30], e.g., pathogens [31, 32], model organisms [33–35], and human [36], among others. The COnstraint-Based Reconstruction and Analysis (COBRA) toolbox [37] has become instrumental in organizing an extensive collection of genome-scale models and analysis tools accessible, and has proven to be a valuable resource to the community [38]. Flux Balance Analysis (FBA) [39, 40] and Flux Variability Analysis (FVA) [41, 42] are two related constraint based modeling approaches for inferring optimal reaction flux rates, or feasible ranges of flux rates, respectively. These approaches rely on an objective function with constraints that enforce network stoichiometry and bounds on the individual fluxes, see [40] for an overview.

There have been several attempts to merge constraint-based models with regulatory constraints. Regulatory FBA (rFBA) [43, 44] and Steady-state Regulatory FBA (SR-FBA) [45] are among the earliest to encode regulatory constraints into FBA using Boolean logic. Integrated FBA (iFBA) [44] and Dynamic FBA (DFBA) [46] connects the FBA framework with kinetic models of metabolism described by ordinary differential equations. Probabilistic Regulation of Metabolism (PROM) utilizes conditional probabilities of gene states (on and off) to model transcriptional regulation [47]. These probabilities are estimated by the frequencies of co-occurrence within the samples, e.g., *P*(*A*=*on*∣*B*=*off*) is an estimate of the number of samples such the target gene *A* is on given transcription factor *B* is off. The effect of a knock out at the genome scale can then be assessed by building the probabilities associated with the target genes into upper bounds for FVA. PROM requires massive sample sizes to stably estimate the probabilities between target regulator pair interactions, and underlying these estimates is the need to discretize the gene expression into on and off states. Transcriptional Regulated FBA (TRFBA) [48] also integrates regulatory and metabolic networks by adding different levels of constraints to bound the rate of reaction supported by a gene, correlation between target and regulating genes to limit associated reaction of a given gene, and finally a set of binary variables is added to prevent overlapping or conflicting constraints. Other approaches have utilized object-oriented modeling, most commonly applied in automotive and process industries, to integrate metabolic and regulatory systems [49–51].

Transcriptional abundance has also been utilized to derive context-specific metabolic models [52, 53]. The underlying rationale is that not all biochemical reactions in a genome-scale reconstruction are active in a given cell type or condition, and refining the model and flux estimation accordingly, will lead to more precise in silico predictions. Methods such as Gene Inactivity Moderated by Metabolism and Expression (GIMME) [54], integrative Metabolic Analysis Tool (iMAT) [55] and Metabolic Adjustment by Differential Expression (MADE) [56] seek to derive context-specific models that are more consistent with measured transcriptional abundance. These approaches rely on thresholding to discretize gene states as active/inactive for high/low expression levels, respectively. E-flux derives maximum flux constraints for FBA from gene expression data with the underlying assumption that mRNA can be used as an estimate of the maximum available protein [57]. Machado et al. evaluated the above approaches to context-specific metabolic modeling on three datasets, and concluded that each approach is relatively comparable in terms of performance, and that there is often no significant gain over standard models of FBA that do not incorporate transcriptomics data [53]. Recently, Least squares with equalities and inequalities Flux Balance Analysis (Lesi-FBA) [58] was developed to predict changes in flux distributions from gene expression changes between diseased and normal brain tissues. Notably, many of the existing methods for predicting fluxes utilizing gene expression are most effective when large changes gene expression changes are observed. In contrast, Lesi-FBA utilizes fold changes in the inequality constraints for the optimization in order to confine the region of feasible fluxes for FVA, and thus does not require discretization. Consequently, Lesi-FBA is more sensitive to subtle changes in gene expression, which alternative methods relying on discretization are too crude to capture.

In this work, we aim to integrate a gene regulatory network into a constraint-based metabolic networks model using probabilistic reasoning as the “glue” that binds these two systems. Specifically, probabilistic reasoning provides an underlying framework for predictions of the system-wide effects of genetic (node) perturbations in a regulatory network [25]. These predicted effects can be quantified and embedded into FVA constraints, thereby constraining the metabolic network with predictions from the gene regulatory network. Both modeling paradigms inherently account for uncertainty in the data and modeling. Our novel approach has the following advantages. The approach (1) does not require discretization of gene expression data, (2) does not require data from more than one experimental condition (e.g., treatment effects, disease/non-disease or knock out), (3) directly accounts for the structure of the gene regulatory network, (4) quantifies and embeds the probabilistic constraints derived from a BN that is parameterized by gene expression data, (5) predicts a range of metabolic states that is within the support of the expression data. This approach is applied to a model of brain metabolism to explore perturbations in the HIF-1 (Hypoxia-Inducible Factor 1) signaling pathway, which has been shown to have protective effects in neurodegenerative disorders [59, 60]. Specifically, HIF-1 is a protein complex that is critical in regulating the body’s response to low oxygen concentrations and hypoxia. Our approach characterizes the effectiveness of perturbations within this pathway on the metabolic state in healthy patients, and those with Alzheimer’s Disease (AD). Our results support HIF-1A as a effective target to reduce the effects of hypoxia, a hallmark of AD. However, the pathway as a target is far less effective in shifting metabolism than in control (healthy) patients. Integrative models predict that HIF-1 activation increases flux through anaerobic glycolysis and ATP production in normal brains. However, this effect was observed to be considerably weaker in AD patients.

## Methods

### Probabilistic modeling: Bayesian networks and probabilisitic reasoning

PGMs are a flexible class of models that encode probability distributions between a set of random variables, *X*={*X*_1_,*X*_2_,…*X*_*p*_}, in the graph that nodes (aka vertices) represent random variables [16, 61]. In our case, nodes represent measured biological variables from an experiment, such as gene expression. BNs are a special class of directed PGMs that are used to describe the direct and indirect dependencies between a set of random variables, and have shown tremendous value in biological applications e.g., [17, 23, 62–66]. In this work, we rely on BNs to model the relationships in a known signaling pathway. There are two major advantages in using BNs in this context: (1) there is a unique mapping between the network and the probability distribution, and (2) exact inference for probabilistic reasoning can be performed.

Briefly we provide an overview of BNs, see [16, 61] for a more comprehensive treatment of the topic. BNs follow the *Markov condition*, which states that each variable, *X*_*i*_, is independent of its ancestors, given its parents in graph, *G*. The conditional independencies between variables (nodes) is depicted in *G*, and can be used to express joint distribution in compact factored form. Under these assumptions, a BN encodes conditional independence relationships:

$$P\left(X_{1},X_{2},\ldots, X_{n}\right) = \prod_{i=1}^{n} P\left(X_{i} \mid {\rm{pa}}(X_{i}), \Theta_{i} \right),  $$

where *pa*(*X*_*i*_) are the parent nodes of child node, *X*_*i*_, and *Θ*_*i*_ denotes the parameters of the local probability distribution. The conditional probability of a child node given its parents, *P*(*X*_*i*_∣*pa*(*X*_*i*_),*Θ*_*i*_), is often referred to as a *local distribution*. In our applications, these local models are Gaussian and are parameterized using gene expression data via local regressions on parent nodes [61].

Probabilistic reasoning in a BN utilizes evidence about nodes in the network in order to reason (query) information about other nodes in the network [61]. In our settings, this *evidence* relates to changes in an upstream transcription factor. The probabilistic reasoning paradigm can be leveraged to predict updated probabilities and states of nodes in the network after taking new evidence into account. Probabilistic reasoning can be viewed as a tool to predict comprehensive system-wide responses of the network to new evidence, which is akin to an in silico experiment. Belief Propagation (BP) algorithms enable the absorption and propagation of evidence through a network [67]. BP in a BN is computed on a junction tree or elimination tree, see [25, 61, 68, 69] for a detailed description.

This work utilizes the BP procedure in the BayesNetBP package, which implements the algorithms described in [69]. The outputs of belief propagation are the predicted parameters for the local distributions in a BN after the absorption and propagation of new evidence into node(s) in the network. Nodes that are d-connected to absorbed node(s) will exhibit changes in their parameters. Comparison of these parameter changes can be used to quantify system-wide effects in the network after evidence is entered, e.g., via fold-changes of mean estimates or Kullback-Leibler divergence [25].

### Constraint-based models of metabolism

Cellular metabolism can be modeled using the principals of mass balance [70] as a system of Ordinary Differential Equations (ODEs):

$$\frac{dC}{dt} = E \cdot \Phi, $$

where *C* denotes the concentration of metabolites, *E*∈**R**^*m*×*n*^ is the sparse stoichiometric matrix and *Φ*∈**R**^*n*×1^ contains the flux rates for the reactions in the model. When the system is at *steady state* the system of ODEs simplifies to a linear system, which is our underlying assumption. The addition of constraints can serve many purposes, e.g., to impose the irreversibility of certain reactions, to add a priori knowledge about flux rates or linear combinations of flux rates. Mathematically, the addition of constraints shapes the solution space for the flux estimation [40]. An objective function can also be used to maximize fluxes or linear combinations of fluxes related to optimal growth conditions, ATP production or a biomass production rate [39, 40].

The objective of FVA is to estimate feasible solutions to the constrained optimization problem [41, 42], which can be described mathematically as follows:

1 $$ \begin{aligned} & \underset{\Phi}{\text{max}} & & c^{T} \Phi \\ & \text{subject to} & & E\Phi = f, \\ &&& G\Phi \ge h, \end{aligned}  $$

where *E*^*m*×*n*^ is the stoichiometric matrix with rows representing *m* metabolites and columns for *n* fluxes, and *Φ* is a vector of fluxes. The concentrations of metabolites does not change under the steady state assumption. External metabolites participate in uptake or release to the extracellular environment, or are not fully accounted for in the model. Therefore, the net fluxes for these external metabolites can be non-zero. The inequality constraint *GΦ*≥*h* can be used to impose irreversibility of certain reactions as well as the capacity constraints that provide the upper limit of fluxes. The objective function, *c*^*T*^*Φ*, is a linear combination of the fluxes that are to be optimized. In our applications, we seek the maximization of net ATP production in the feasible space of *Φ*, because the brain has a very high requirement on energy production, which is critical for bioenergetics, function and neurodegeneration [71]. This objective function was also used in the model developed by Gavai et al. [58]. Equality constraints can be used to encode uncertainty in the fluxes, which can be leveraged in sampling, or when additional constraints are present, such that no solution to the linear system exists. Let *b* represent the measured fluxes and *ε* be the measurement errors, then the observation model is given as:

2 $$ \left.\begin{aligned} E\Phi &= b + \epsilon,\\ \end{aligned}\right.  $$

while still satisfying the constrains in Equation 1.

### Computational model of the brain

***Model of brain metabolism:*** A core metabolic model for normal human brain was constructed using 89 metabolites, 71 biochemical reactions from core pathways, including the glycolytic pathway, Pentose Phosphate Pathway (PPP), the TriCarboxylic Acid (TCA) cycle, malate-aspartate shuttle, the glutamate and GABA shunt and oxidative phosphorylation. The model spans the extracellular space, cytosol and mitochondria. This core model was originally used to investigate the low oxygen to carbohydrate ratio in the brain during extreme endurance sports [72], and later used to examine to characterize the metabolic changes in Alzheimer’s patients [58]. These investigations, including our own, utilize flux estimation of the metabolic model at steady state. A full description of the models biochemical reactions is given in Additional file 2: Table S1.

***Bayesian Network of the HIF-1 signaling pathway:*** The structure of the BN is constructed from the HIF-1 signaling pathway in the KEGG database [73]. The R packages graphite [74] and pcalg [75] were used to create the network and transform it into a directed acyclic graph. Specifically, the cyclic structure and bidirectional edges were eliminated through the construction of a partially oriented graph, see [76] for details. This method directs the undirected edges without creating cycles in the graph. This is critical because cycles (aka feedback loops) in the graph are prohibited in order to make the factorization of the likelihood tractable [61]. This approach also does not induce additional v-structures *A*→*C*←*B*, which would create additional independencies in the graph. The full network consists of 86 nodes and can be viewed in Additional file 1: Figure S1. In order to connect the probabilistic (genetic) to the constraint-based (metabolic) models, member of the genes in the HIF-1A pathway were mapped to the enzymes in the metabolic model. A total of 15 genes mapped to enzymes in the metabolic model (Additional file 2: Table S1) and they are concentrated in the glycolysis pathway.

Two BNs were constructed with the identical structure of the signaling pathway (Additional file 1: Figure S1). However, these networks were parameterized differently by using data from brain gene expression data from healthy and Alzhiemers Disease (AD) patients brains. The microarray data used in this study was taken from the Gene Expression Omnibus with the accession ID GSE5281 [77, 78]. This dataset contains gene expression measurements from laser captured micro dissected neurons from healthy and AD subjects. For the present analysis, only the hippocampus region is utilized, which is the region most affected during the early stages of the disease. These parameterized models were used to investigate the effects of up-regulated HIF-1A on the expression of other genes using probabilistic reasoning via belief propagation on the HIF-1 pathway. Evidence for the HIF-1A transcription factor was absorbed at six different values of transcript abundance levels over the range of 8 to 13. Therefore, the belief propagation algorithm was applied six times, once for each absorbed piece of evidence. This was performed for both the control and AD models. For each absorbed evidence, the fold-changes of d-connected nodes were estimated. For the calculation of the predicted fold-changes, the mean expression level of the gene of interest in the original data set was used as the denominator, while the mean expression level after HIF-1A perturbation, as obtained through BP, was used as the numerator.

**Fig. 1:**
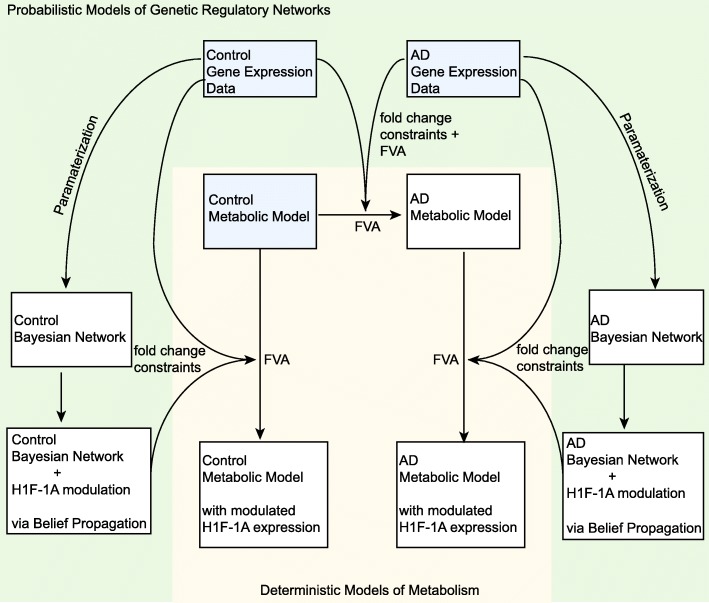
A schematic of the interface between the probabilistic model of gene regulatory networks (green) and constraint-based models of metabolism (yellow). Light blue boxes indicate core models and data. White boxes correspond to predicted models. Control and AD gene expression is used to characterize metabolic states via FVA (flux variability analysis) in a control metabolic model of brain metabolism and an AD metabolic model of brain metabolism. The BN is used to predict the enzymatic responses of enzymes in the model after HIF-1A modulation in control and AD models using belief propagation. These predicted enzymatic responses are used to constrain the FVA in the control and AD metabolic models

***Interfacing the metabolic and signaling models:*** The AD metabolic model at HIF-1A basal level is obtained using Lesi-FBA [58]. The interface between the metabolic model and the BN representation of the signaling pathway is created through the use of BP-based constraints on the metabolic model (Fig. 1). Different sets of constraints were formed using information from the respective instance of BP in the two BNs. Each BP procedure produces a set of estimated fold changes, which can be embedded into the the constraints (Equation 2). Specifically, BP results are used to predict fold changes of enzymes in the biochemical reactions, and the fluxes from the initial model are scaled by the fold-changes. The predicted constraints for the fluxes are embedded into *b* in Equation 2. In cases where multiple enzymes mapped to a single reaction, the average fold-change across these genes was used to constrain the corresponding flux. This enable us to capture the fold-change of an enzyme even if their abundance is small, which can be important in regulating a reaction. The implicit and simplifying assumption of these derived constraints is that the reaction rates change in a way that is proportional to the enzymatic changes in the model reflected by mRNA expression. This approach has also been adopted by Gavai et al. [58]. Note that the variance for the local distributions for the BN models after BP is not directly amenable to the constraints in the metabolic model. Variance estimates for the enzyme constraints were estimated from the model with no use of gene-expression data, using the methods of [58] that are based on measured uptake and release rates [79]. The estimates were used as input into the metabolic model and FBA was performed to estimate the variances of *ε*. Thus, no gene expression data was used in the variance estimation.

Another constraint was formed using knowledge about the pyruvate dehydrogenase (PDH) regulation, which is a connection between glycolysis and the TCA cycle. Pyruvate dehydrogenase kinase 1 (PDK1) is a known downstream target of HIF-1 regulation, which can inactivate PDH through phosphorylation [80], a post-translational modification. Therefore, in addition to its expression fold-change, the activity of PDH further depends on PDK1 expression. Since PDH is a key enzyme of TCA cycle, we took this effect into account by further multiplying the predicted fold-change of PDH by 1/*α*, where *α* is the predicted fold change of PDK1 from belief propagations with different values of HIF-1A.

Taken together, ten constraints were added to the model. The system of equations is overdetermined, and thus the solution is not unique. The least square solutions of *Ax*=*b*+*ε* was computed using the *lsei* (Least Squares or quadratic programming problems under Equality/Inequality constraints) routine in the R LIM package [81, 82]. FVA was then performed in R using the *mirror* algorithm that is implemented in the *xsample* function [83]. The function *xsample* implements Markov Chain Monte Carlo (MCMC) sampling to uniformly sample the feasible region of the constrained optimization problem. The *mirror* algorithm for MCMC takes advantage of *reflections* that are guided by the inequality constraints, which improves acceptance rates and mixing for the chain when compared to hit-and-run samplers [83]. FVA models were fit for each value of HIF-1A that was absorbed into the signaling network in order to generate a new set of constraints. In total, six sets of constraints were generated for each condition, and 12 FBA models were fit. This analysis was performed for both the control and AD datasets. The convergence of the MCMC was assessed using the approaches of Gelman [84] and Geweke [85]. Specifically, the Geweke statistic is based on a test for equality of the means of the first and last part of a Markov chain (the first 10% and the last 50%). If the samples are drawn from the stationary distribution of the chain, the two means are equal and Geweke’s statistic has an asymptotically standard normal distribution. The Gelman diagnostic compares the pooled variance of multiple chains with the variances of each chain and will approach one if the Markov chain converges. The code for this analysis was written in the R programming language, and is available at code https://github.com/hyu-ub/prob_reg_net.

## Results

**Fig. 2:**
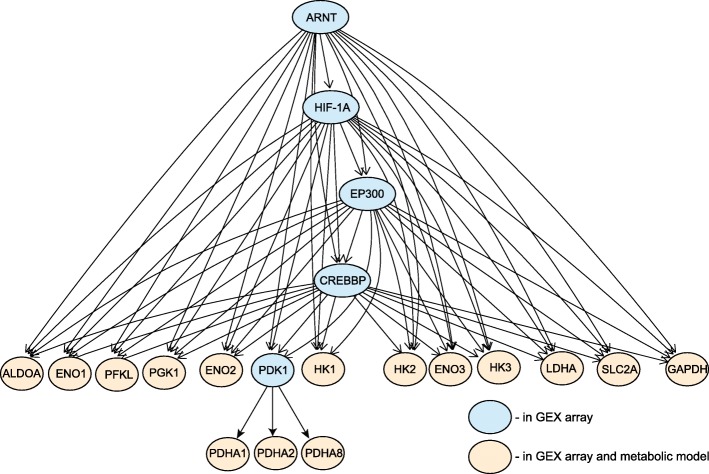
Schematic of select nodes in the HIF-1A sub-pathway. The sub-pathway includes the enzymes in the metabolic model and their ancestors

The integrated model consists of a signaling pathway represented by a BN and a constraint-based model of cellular metabolism in the brain. These models are interfaced through belief propagation (Fig. 1), which enables prediction for the network under perturbation, and is used to constrain the FVA for the steady state estimation of fluxes in the metabolic model. A model for the HIF-1 signaling pathway was constructed using a BN approach, and parameterized using AD and control data (Fig. 2 & Additional file 1: Figure S1). The BN for the pathway was parameterized with gene-expression data from control and AD patients. In the gene expression data, the mean abundance level of HIF-1A is 9.29 in control group and 9.65 in the AD group.

**Fig. 3:**
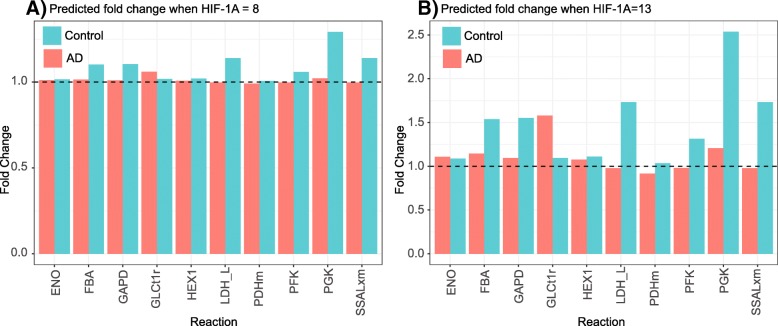
The predicted fold change of enzymes in the network after absorbing and propagating evidence into HIF-1A for control (blue) and AD (red) patients. The expression level was varied between 8 and 13 for the modeling. The predicted fold change at the extreme values is shown, i.e., **a** when HIF-1A was set at 8, and **b** when the expression level of HIF-1A was set at 13

Enzyme abundance levels were estimated for control and AD models when ranging HIF-1A between low (8) and high (13) levels. These estimated abundance levels were subsequently utilized to derive fold changes between estimated basal and repressed/activated levels of HIF-1A for control and AD models. The predicted fold changes for the lowest (Fig. 3a) and highest (Fig. 3b) levels, indicate large changes with high HIF-1A abundance, particularly in control samples. This suggests that the metabolism in the control model will be more sensitive to HIF-1A perturbations when compared to the AD model.

Estimated fold-change constraints were derived from belief propagation for both the control and AD models. These constraints were utilized for the inequality constraints for the FVA. Taken together, this leads to a total of 12 FBA models that correspond to six different levels of HIF-1A in control and AD BNs. For each of these models, MCMC was run for 100,000 iterations and the first 2000 were disregarded as burn in. MCMC diagnostics indicated convergence (Additional file 1: Figure S2). The estimated fluxes for all reactions in the model when HIF-1A is at basal level (HIF-1A expression = 9.5) and strongly activated (HIF-1A expression = 13) are given in Additional file 2: Table S1. Overall, the estimated fluxes for the AD model was far less sensitive to changes in HIF-1A levels.

**Fig. 4:**
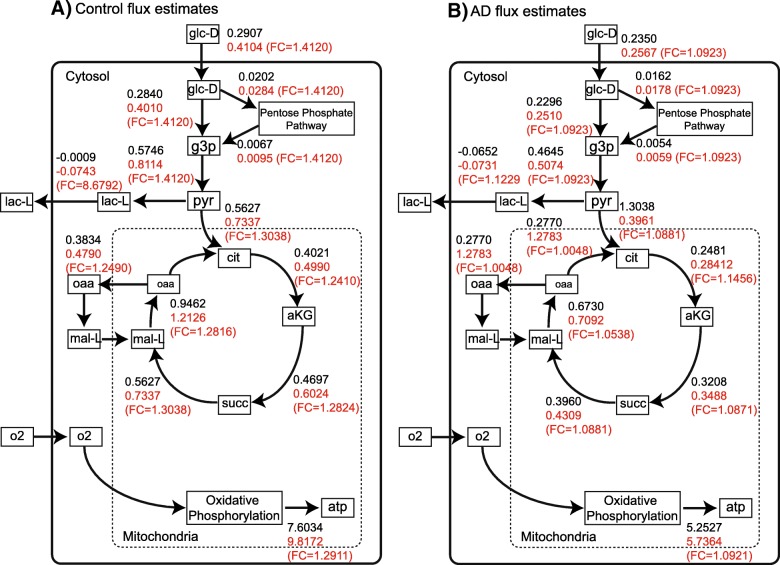
Simplified schematic of the constraint-based model of cellular metabolism. Detailed estimates for all reactions in the model are given in Additional file 2: Table S1. For each reaction in the model, the predicted flux estimate based on belief propagation constraints that were derived by setting evidence of HIF-1A expression is 8 (top number), increasing HIF-1A expression levels to 13 (middle number), and the fold change (bottom number). These flux estimates are displayed for the **a** control data, and **b** AD data

**Fig. 5:**
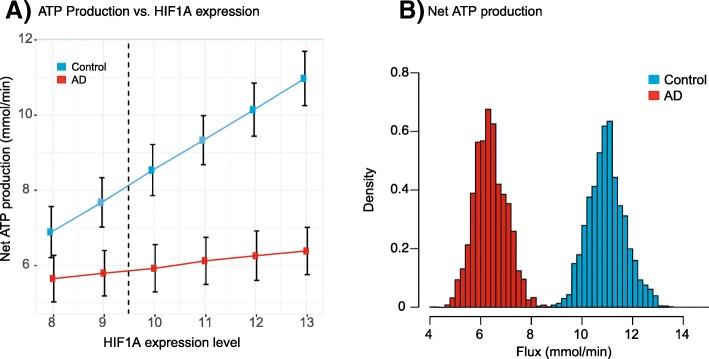
Results from the ensemble of flux estimations. (A) Predicted rate of ATP production as a function of HIF-1A expression, which was set to range from 8 to 13. The mean and standard errors are shown for the control group (blue) and the AD group (red). The dotted line indicates the basal level of 9.5. (B) Histograms of predicted ATP production for the model for the control group (blue) and the model for the AD group (orange) when HIF-1A is 13

A simplified schematic of the flux rates for the core energy metabolism is shown for control data (Fig. 4a) and AD data (Fig. 4b). The BP-based estimate of relative fold change of fluxes within the AD and control groups for each reaction is also indicated. Overall, HIF-1A up-regulation increases fluxes in glycolysis and the TCA cycle. However, this increase considerably larger in control samples. Our estimates also suggest that the majority flux changes were smaller in the AD model when compared to the control model (Fig. 3), this is more apparent as the level of HIF-1A is increased (Fig. 4 & Additional file 2: Table S1). The majority of these reactions belong to the glycolysis pathway, including the rate-limiting reaction facilitated by phosphofructokinase (R_PFK: PYR →CIT). The changes in flux distributions also showed a major impact on the predicted rate of net ATP production (Fig. 5). When HIF-1A expression was increased from 8 to 13, ATP production also increases, but to a lesser degree in the model using AD samples. Therefore, ATP production was shown to be more sensitive to HIF-1 pathway activation in control models. Consequently, this suggests that the activation of HIF-1 pathway is less efficient in terms of remedying ATP reduction in AD brains.

HIF-1 activation in control model enhanced the energy production through anaerobic glycolysis by more than 8-fold, while that from TCA cycle increased only by 30%. Although the oxygen consumption also showed an increase, the overall trend of shifting flux from TCA cycle to anaerobic glycolysis is consistent with the known function of HIF-1 pathway. One the other hand, such effect is much weaker in AD models.

## Discussion

In this work, we developed an approach to integrate probabilistic graphical models of gene regulatory networks into constraint-based models of metabolism. An in silico model of this type can provide novel insights into potential therapeutic targets that may be otherwise costly, time-consuming or experimentally prohibitive. Utilizing a BN framework enables parameterizations using gene expression data, and probabilistic queries to the network to derive constraints for flux estimation in the metabolic model. In this context, probabilistic reasoning via belief propagation actually re-casts the BN as a computational model that can be used to derive constraints for the FVA. To the authors knowledge, this is the first approach to integrating gene regulatory networks parameterized by gene expression into steady state models of metabolism that does not require boolean logic, thresholding, massive sample size or classic treatment/control type experiments. Our approach is comparable to lesi-FBA, which utilizes fold-changes from the gene expression in the FVA constraints [58]. In fact, the AD metabolic model (Fig. 1) was estimated using this approach, and reproduces the results in Gavai et al. at basal level [58]. However, in contrast to lesi-FBA, our approach leverages the BN as a computational model for probabilistic reasoning in order to generate predicted fold-changes for various perturbations and conditions. Thus, our approach can perform in silico predictions of how the metabolic state shifts under perturbation to the gene regulatory network.

AD is a neurodegenerative disorder characterized by severe memory and cognitive function impairment. Although the underlying molecular mechanisms are not fully understood, hypoxia has been implicated in the pathogenesis and progression of AD [86, 87]. Hypoxia-inducible transcriptional factor-1 (HIF-1) is a major controller of the hypoxic responses associated with neurodegenerative disorders [88]. However, conflicting evidence regarding its role in AD exist, and manipulation of the hypoxic pathways can have different outcomes [60]. There has been some positive evidence surrounding HIF-1 activation as a strategy to slow the progression of AD [59, 89, 90]. For example, HIF-1 target gene EPO has also been shown to have protective effects and has been considered for potential AD treatment [91, 92]. Our novel approach was utilized to predict the metabolic states over a range of HIF-1 levels in a constrain-based model of brain metabolism. HIF-1 is known to promote cellular responses to reduced glucose supply, low oxygen levels and oxidative stress. Specifically, activation of HIF-1 pathway has been known to increase glucose uptake, glycolysis, and the conversion of pyruvate to lactate, by which ATP production is maintained even in oxygen deprivation.

Prediction from the model estimate an 8-fold increase in anaerobic glycolysis in control brain cells when HIF-1A level is increased to 13 from 8, which is consistent to the known HIF-1 function. However, this effect is much weaker in AD brains. Under the same conditions, the increase in fluxes in glycolysis pathway and TCA cycle are only around 10%. This result suggests HIF-1 in AD is less efficient in modulating energy production by directly regulating enzyme activities. This could be due to the fact that in AD the anaerobic glycolysis level is already high at HIF-1 basal level. On the other hand, HIF-1 may still remedy energy depletion through other mechanisms, such as erythropoiesis and angiogenesis, which can not be quantified by our models. Taken together, our results are physiological and support HIF-1A as a potential target for AD patients. However, our models suggest that the target will not elicit the same degree of metabolic response that would be present in a control (healthy) brain. Considering the side effects of HIF-1 activation, and its lower efficiency in rescuing deficient energy production, HIF-1 pathway is perhaps not an ideal therapeutic target for AD patients. Therefore, the therapeutic benefit of HIF-1 activator in AD patients is probably not through directly modulating intracellular energy metabolism. If data becomes available, it would be informative to reproduce this in silico experiment to characterize AD brains in early and late stage AD patients, as it is expected that the metabolic shift from healthy patients is more subtle in early-stage [93–95]. Thus, we hypothesize that HIF-1A may be most effective in early-stage patients.

There are several limitations in this approach that are inherited from the underlying representations of the gene regulatory and metabolic networks. Notably, the gene regulatory network is integrated into a metabolic network, and the modeling framework does not allow for other way around. Thus, the one-way integration of networks describes the impact of the genetics on metabolism [96], but will not capture metabolism effects on gene regulation [97]. Furthermore, BN does not have cycles, and thus do not provide the flexibility that an undirected graph with cycles (Markov Network) would provide for modeling gene regulatory networks [61]. Despite this limitation, in many cases, directed acyclic graphs have been shown to capture nonlinear and feedback behaviors reasonably well [65]. Moreover, undirected graphs do not provide an infrastructure for exact inference, and thus do not lend themselves to reliable predictions for the estimated fold constraints that are embedded into the FVA.

Limitations outlined in Blazier et al. [52] that arise from connecting gene expression to the metabolic model, are also inherent in our models. For example, crude summarizations via averaging of the enzyme activity were utilized when multiple enzymes and/or isoforms regulated a reaction in the metabolic model. BNs were also parameterized using only transcriptional gene expression data from bulk tissue samples from the hippocampus [78], which does not capture critical activities such as protein degradation or post-translational modification. It has also been shown that the degree of correlation between gene expression and protein data is rather weak [98]. Taken together, these data are limiting and likely a poor surrogate for neuronal activity. At present, to the authors knowledge, there are no publicly available protein datasets or single cell datasets, from human AD and control brains. However, the model can and will be easily modified as additional protein and single cell data sources become available.

In conclusion, the integration of probabilistic graphical models of gene regulatory networks into constraint-based models of metabolism networks provides a unique opportunity to assess the impact of in silico genetic perturbations to downstream metabolism. Moreover, leveraging probabilistic reasoning facilitates predictive modeling of enzymatic activity that extends beyond the gene expression data. Future work will be extending this paradigm to genome-scale models [99]. In order to achieve this, an undirected PGM could be leveraged in place of a BN. However, as described above, the probabilistic reasoning via belief propagation is only approximate in this case, whereas it is exact for BNs [61]. Properly accounting for this approximate inference in a scalable manner will be an area of future research.

## Additional files

Additional file 1 Supplemental figures. (PDF 110 kb)

Additional file 2 Supplemental table. (XLSX 17 kb)

## Abbreviations

AD: Alzheimer’s Disease
BN: Bayesian Network
BP: Belief Propagation
COBRA: COnstraint-Based Reconstruction and Analysis
DFBA: Dynamic Flux Balance Analysis
FBA: Flux Balance Analysis
FVA: Flux Variability Analysis
GIMME: Gene Inactivity Moderated by Metabolism and Expression
HIF-1: Hypoxia-Inducible Factor 1
iMAT: integrative Metabolic Analysis Tool
Lesi-FBA: Least squares with equalities and inequalities Flux Balance Analysis
MADE: Metabolic Adjustment by Differential Expression
MCMC: Markov Chain Monte Carlo
ODE: Ordinary Differential Equation
PDH: Pyruvate Dehydrogenase PPP - Pentose Phosphate Pathway
PGM: Probabilistic Graphical Model
PROM: Probabilistic Regulation of Metabolism
rFBA: regulatory Flux Balance Analysis
SS-FBA: Steady-state Regulatory FBA
TCA: TriCarboxylic Acid
TRFBA: Transcriptional Regulated FBA
