# Supplementary material for: Integration of probabilistic regulatory networks into constraint-based models of metabolism with applications to Alzheimer’s disease
Source: BMC Bioinformatics. 2019 Jul 10;20:386. doi: 10.1186/s12859-019-2872-8 (PMC6617954; doi:10.1186/s12859-019-2872-8)
Supplement: Supplementary file 1 — Supplemental figures. (PDF 110 kb) [file 12859_2019_2872_MOESM1_ESM.pdf]

Supplemental Figures

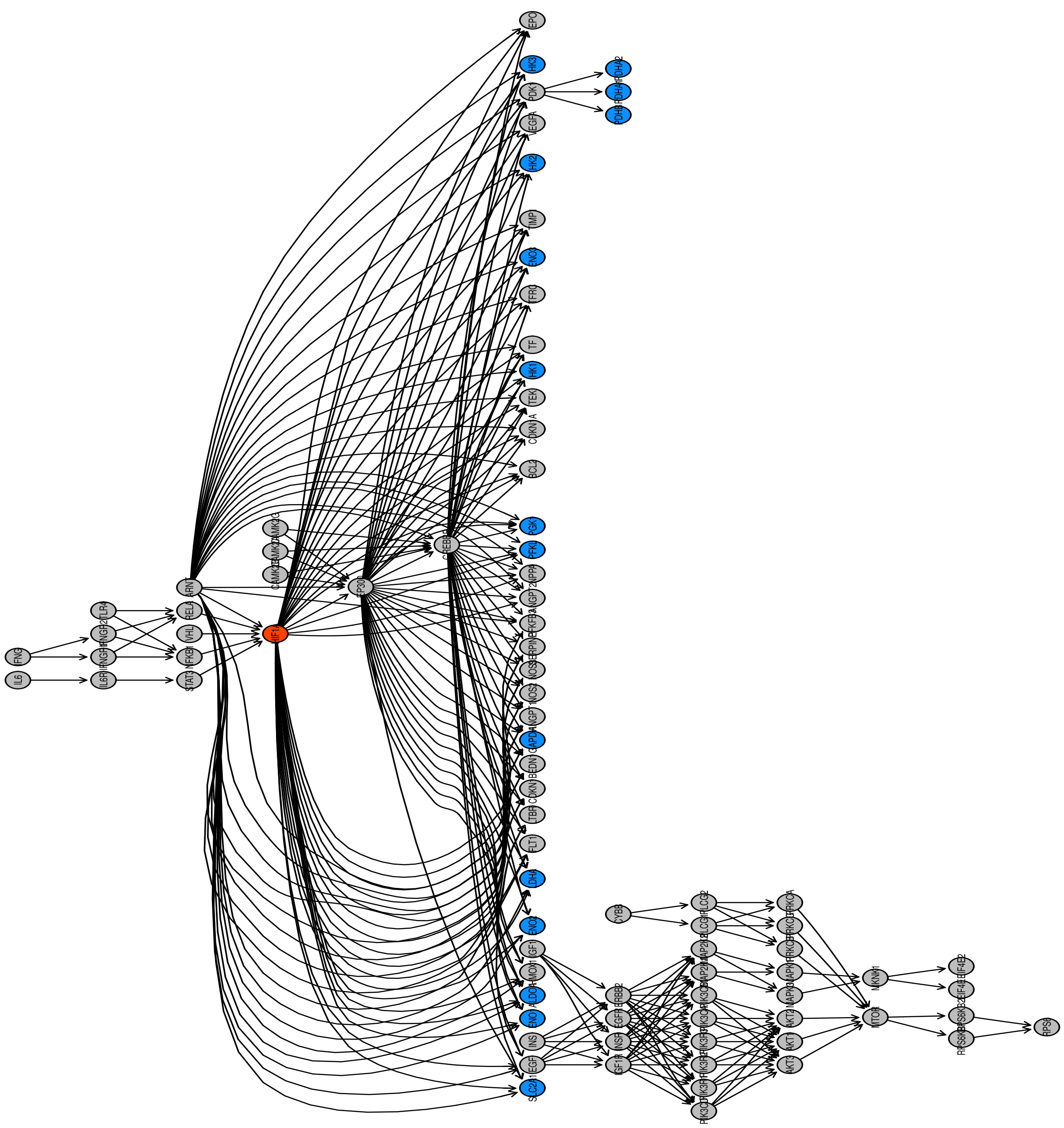

Figure 1: The full H1f-1 signaling pathway consists of 86 nodes. The nodes for which gene expression data is available are colored in blue. The H1F-1A transcription factor is colored in red.

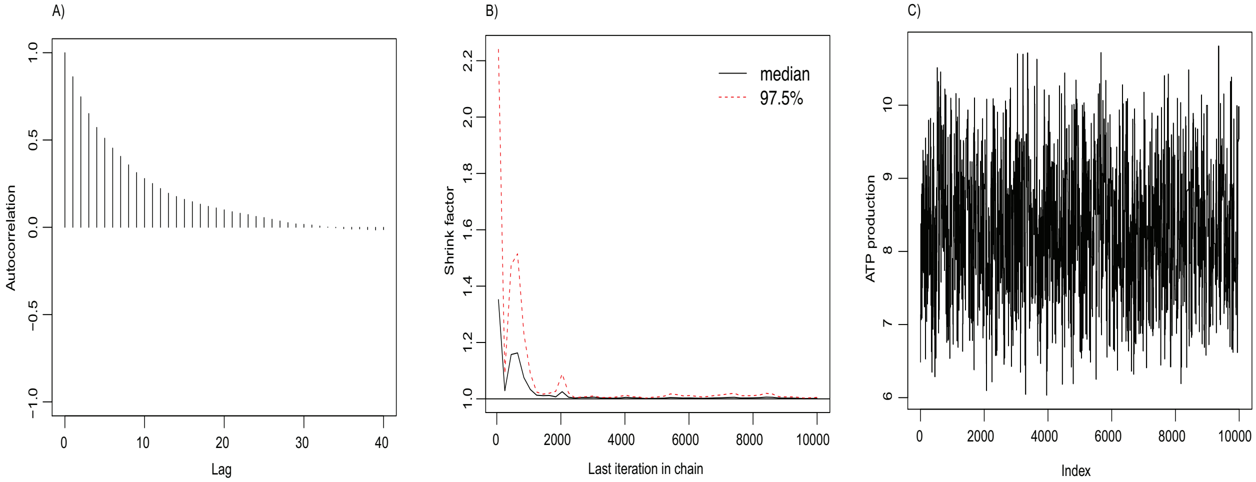

Figure 2: (A) The autocorrelation of a MCMC chain, (B) the Geweke statistic and (C) the predicted flux of ATP production across the MCMC sample. The chains for other models exhibited similar behavior, suggesting convergence.
